# Watch and Wait Approach for Rectal Cancer

**DOI:** 10.3390/jcm12082873

**Published:** 2023-04-14

**Authors:** Carlos Cerdan-Santacruz, Guilherme Pagin São Julião, Bruna Borba Vailati, Leonardo Corbi, Angelita Habr-Gama, Rodrigo Oliva Perez

**Affiliations:** 1 Department of Coloproctology, Hospital Universitario de la Princesa, 28006 Madrid, Spain; 2 Department of Coloproctology, Clínica Santa Elena, 28003 Madrid, Spain; 3 Angelita and Joaquim Gama Institute, São Paulo 01329-020, Brazilbrunabvailati@gmail.com (B.B.V.); leonardo.corbi@hotmail.com (L.C.); gamange@uol.com.br (A.H.-G.); 4 Department of Coloproctology, Hospital Alemão Oswaldo Cruz, São Paulo 01323-020, Brazil; 5 Department of Surgical Oncology, Hospital Beneficencia Portuguesa, São Paulo 01323-001, Brazil

**Keywords:** rectal cancer, clinical complete response, watch and wait, organ preservation, total neoadjuvant therapy, immunotherapy, local recurrence, local tumor regrowth, near-complete response

## Abstract

The administration of neoadjuvant chemoradiotherapy (nCRT) followed by total mesorrectal excision (TME) and selective use of adjuvant chemotherapy can still be considered the standard of care in locally advanced rectal cancer (LARC). However, avoiding sequelae of TME and entering a narrow follow-up program of watch and wait (W&W), in select cases that achieve a comparable clinical complete response (cCR) to nCRT, is now very attractive to both patients and clinicians. Many advances based on well-designed studies and long-term data coming from big multicenter cohorts have drawn some important conclusions and warnings regarding this strategy. In order to safely implement W&W, it is important consider proper selection of cases, best treatment options, surveillance strategy and the attitudes towards near complete responses or even tumor regrowth. The present review offers a comprehensive overview of W&W strategy from its origins to the most current literature, from a practical point of view focused on daily clinical practice, without losing sight of the most important future prospects in this area.

## 1. Introduction

Management of rectal cancer has considerably changed over the last few decades [1]. While radical surgical resection with total mesorectal excision (TME) remains as one of the pillars of treatment, introduction of multimodality therapy with radiation and chemotherapy was fundamental for the development and proposal of organ-preservation strategies [2]. Initially used to improve local disease control, radiation alone or chemoradiation used in the preoperative (neoadjuvant) period was shown to be more effective than postoperative (adjuvant) treatment [3]. In addition to improvements in local disease control, preoperative treatment resulted in variable degrees of primary tumor response [4]. Observation of complete disappearance of the primary tumor (clinical complete response—cCR) led surgeons to consider avoiding immediate resection in select patients [5]. Since the initial reported outcomes of this non-operative treatment for patients who achieved a cCR, many changes have developed in terms of baseline assessment, neoadjuvant treatment regimens, assessment studies and timing of assessment. In addition, as long-term data become available, there is more information available regarding the risk of local regrowth of tumors which achieved a cCR and also regarding the risk of subsequent distant metastases development [6]. Finally, molecular markers have been able to distinguish a specific subtype of rectal cancer where a distinct treatment alternative may lead to an opportunity for organ-preservation in a significant proportion of cases [7].

## 2. Rationale

The reason why non-operative treatment of rectal cancer is attractive is related to the fact that TME surgery is associated with significant morbidity, mortality and functional consequences [8,9]. Significant disturbances associated with urinary and sexual function have been reported [10,11]. However, most relevant are the consequences associated with bowel function after surgery [12]. Depending on the level of the tumor in the rectum, level of the anastomosis and the requirement for partial or total intersphincteric resection, patients may experience variable levels of symptoms associated with Low Anterior Resection syndrome [13,14,15]. Finally, the requirement of a temporary or definitive stoma may be very critical to many of these patients and avoiding it remains an important, if not the most important, patients’ expectation from rectal cancer treatment. Here, it should be considered that many patients who undergo primary anastomosis for rectal cancer with a temporary stoma may ultimately develop a failed anastomosis. Reasons for a failed anastomosis may include leaks, stenosis, recurrence and poor function. Therefore, a number of patients thought to have received a temporary stoma are ultimately faced with a definitive stoma. Over time, the rate of definitive stomas may be nearly triple initial estimates [16,17].

Non-operative treatment obviates many of these issues by avoidance of TME and therefore exposing patients to virtually no morbidity or mortality. However, caution should be taken as a proportion of patients entering a watch and wait (W&W) protocol will go on to develop a local regrowth of the primary tumor and therefore will require surgical resection [18,19]. This means not all patients with a cCR will avoid surgery. Second, even though functional outcomes among patients undergoing W&W are clearly and far better than TME or even local excision [20], function may ultimately not be as perfect as one would expect or hope [21]. Interesting data suggest that functional outcomes of patients undergoing W&W are not necessarily perfect, possibly due to the effects of radiation therapy to the rectum and anal sphincters [22]. The few studies that compared bowel function and quality of life between patients undergoing W&W and TME surgery were performed in at least 2 retrospective series. Both of them compared patients undergoing W&W to matched–controls among patients with incomplete clinical/pathological response followed by TME [20,21]. The results of these studies using different assessment tools were similar, suggesting superior functional and QoL outcomes for the W&W patients. However, one of the studies suggested that nearly one-third of patients within the W&W group reported severe LARS scores, suggesting potential long-term detrimental effects of RT to bowel function [20]. Altogether, these findings should be interpreted with caution. In addition to the limitations inherent to the studies’ designs, one must consider that severe/poor LARS scores have also been reported by healthy individuals [23]. Finally, the comparison of W&W to TME patients may have compared apples to oranges. Ultimately, patients undergoing TME for residual disease would otherwise have never been candidates for W&W. This suggests that the control group may have been inappropriate for such comparison, with introduction of significant potential selection biases related to tumor response [24]. These issues should be taken into account when counseling patients for organ-preservation with W&W.

## 3. Baseline Features & Selection Criteria

### 3.1. Primary Reason for Neoadjuvant Therapy

Assessment of baseline features is critical for the selection of patients who are candidates for W&W. The initial experience of patients undergoing this strategy was accumulated before the availability of accurate staging tools [2,5]. Magnetic resonance imaging (MRI) was largely unavailable and not standardized. At that time, most patients with rectal cancer were considered for W&W based on response to therapy. This means that selection of patients was based on the final result rather than an intended outcome. Ultimately, patients were undergoing neoadjuvant chemoradiation for oncological purposes—to improve local disease control—and, by accident or chance, achieved a cCR. This was perhaps the only selection criterion at that time.

Progressively, staging modalities evolved over time and MRI interpretation became standardized, allowing for the stratification of patients and tumors based on risk factors for local and distant failure [25,26]. This led to a clearer distinction between patients with high-risk features—requiring nCRT for oncological purposes—and those with low-risk features—where perhaps the only benefit of nCRT would be the achievement of a cCR in an attempt to avoid TME surgery [27,28]. Even though these two scenarios have been referred to as an “accidental” versus an “intentional” organ-preservation approach, one could argue that W&W is never truly “intentional” as guaranteed achievement of cCR is not yet possible. Instead, the difference between the 2 scenarios is that the latter relates to the concept that the sole reason or sole benefit for the use of neoadjuvant therapy is achievement of cCR.

### 3.2. Baseline Features

In addition to the primary reason for the use of nCRT among these patients, assessment of baseline features is relevant for some additional reasons:

First, as findings consistent with cCR are largely subjective, it is highly recommended that comparable methods are used both at baseline and at the time of reassessment [29]. In this setting, detailed characterization of clinical, endoscopic and radiological features at baseline may be critical for the assessment of post-treatment response and identification of cCR or incomplete response [30].

Second, response to therapy seems to be a time-dependent phenomenon and not all patients achieve a cCR at the same time [31,32]. There are data to suggest that larger tumors take longer to respond. In this setting, it becomes relevant to understand the magnitude of response at each reassessment round. This is only possible when reassessment findings are comparable to baseline features. Ultimately, despite being largely subjective, quantification of response is an important piece of information. This is only possible when baseline assessment provides detailed information using the same tools expected to be used in reassessment rounds [33].

Finally, an important feature relates to the position of these tumors in the rectum. Since the beginning, the original description of W&W was restricted for tumors accessible to the finger during digital rectal examination (DRE), usually at an average of 7 cm from the anal verge, measured by rigid proctoscopy [5]. The reasons for these selection criteria relate to the risk-benefit balance. Tumors above/beyond the reach of the finger are probably best treated by TME surgery with far fewer functional consequences, less morbidity and lower risk of definitive stomas. Instead, distal tumors are at considerably higher risk for these negative functional outcomes and stoma requirements [34]. In the setting of risk of local regrowth and subsequent distant metastases, the authors believe that tumors located above this 7 cm distance are more likely to have risks that outweigh benefits of avoiding TME.

**Figure 1:**
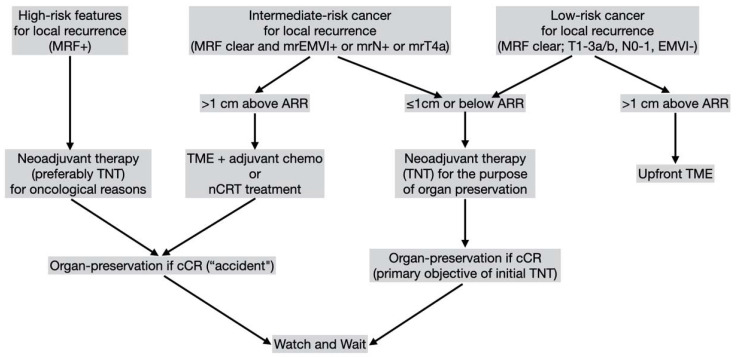
Algorithm for decision management based on risk factors for local recurrence and primary objective to achieve a cCR. ARR: anorectal ring; MRF: mesorectal fascia; mr: magnetic resonance; EMVI: extramural venous invasion; TNT: total neoadjuvant therapy; cCR: clinical complete response; TME: total mesorectal excision. While patients may undergo neoadjuvant therapy for different reasons, decision to W&W is based on the achievement of a cCR. Patients not achieving a cCR are usually recommended for surgical resection (most frequently TME).

As MRI became widely available, in addition to being within the reach of DRE, tumors should be ideally located at the level of or below the insertion of the levator muscles in the pelvis. Tumors with the distal border located 1 cm above/beyond this anatomical landmark are in most cases best suited for TME with or without nCRT, indicated for oncological reasons [34] (Figure 1).

### 3.3. Putting It All Together

Ultimately, baseline assessment is critical for the selection of patients that are being considered for W&W. The following necessary steps may be required before organ-preservation is even a consideration for these patients:

First, clinical (DRE) and endoscopic assessments should provide clear evidence of invasive cancer rather than benign polyps or lateral spreading tumors amenable to endoscopic/endoluminal resections [35]. The finding of mobile and soft lesions at DRE combined with clear endoscopic pit pattern classification may provide precise identification of patients that are candidates for endoscopic/endoluminal resection instead of neoadjuvant therapeutic approaches.

Second, once clinical and endoscopic findings of invasive cancer are present—beyond the scope of endoscopic/endoluminal resection—obtainment of endoscopic biopsies is mandatory. In addition to confirmation of adenocarcinoma histology, tumors are currently expected to be routinely tested for microsatellite instability, preferably through immunohistochemistry, for the presence/absence of the most frequently affected mismatch repair protein/genes [7,36].

Third, magnetic resonance is mandatory for proper staging of these patients and stratification into low-/high-risk categories. While several 2- and 3-tiered categories are available, our approach has been to clearly distinguish patients at high-risk for local recurrence based on mesorectal fascia (MRF) status (threatened when tumors are ≤1mm from the MRF) [28,37]. While tumors with extramural venous invasion (EMVI), lymph node metastases, tumor deposits and T4a may also be considered as high-risk; it is, as of yet, unclear whether these features are independently associated with the risk of local recurrence when there are unthreatened margins [28]. Therefore, MRF+ or positive circunferencial resection margin (CRM+) are patients where neoadjuvant therapy is clearly necessary and organ-preservation may be an additional benefit from this approach (in addition to improved local disease control in the event TME is required). The presence of any high-risk feature does not necessarily exclude the possibility of W&W once a cCR is achieved. This includes the presence of adverse pathological features such as high-grade adenocarcinoma or mucinous tumors (both in pathology and MRI imaging) frequently seen in baseline assessment. However, there is one exception to this rule: the presence of adverse features beyond the limits of the radiation field are clearly an exclusion criterion. The presence of nodal metastases, EMVI or tumor deposits above the cranial limit used for the radiation field should prompt post-treatment surgical resection in most patients, despite complete primary tumor response.

Finally, tumor location and distance from anal verge (by DRE and proctoscopy) and from the insertion of the levator ani (by MRI) are very relevant information to be taken into account to consider patients for W&W in the event of the achievement of a cCR [34] (Figure 1).

## 4. Preferred Treatment Strategy

Once patients have been properly assessed at baseline, one should be able to distinguish patients that are undergoing neoadjuvant therapy for sole purpose of achieving a cCR (distal and low-risk) from those that need therapy due to the high-risk of local recurrence (distal and threatened MRF) [28,37]. In both situations, maximal response is required to achieve a cCR and/or R0 resection (if surgery is ultimately required).

Maximal primary tumor response has been investigated in multiple studies, mostly by complete pathological response (pCR) as a surrogate for cCR [38,39].

Originally, the earliest experiences with cCR and W&W were derived from series using neoadjuvant chemoradiation (nCRT) with long-course radiation (RT) and concomitant 5FU-based chemotherapy [5]. While oxaliplatin has been associated with slightly higher pCR rates when used in combination with 5FU during RT, the significantly higher toxicity rates have limited its use in clinical practice [40,41]. As mentioned previously, response to treatment in rectal cancer after nCRT is a time-dependent phenomenon. In this setting, many systematic reviews and meta-analyses suggested that longer interval periods between the end of radiation and surgical resection were associated with higher rates of pCR [42,43]. This led to the consideration of the use of short-course RT followed by prolonged intervals (instead of the classic 1-week interval to TME) as an alternative to achieve similar rates of pCR as compared to standard nCRT using long-course RT [44]. While this did result in higher pCR rates, experience with cCR and W&W was largely unavailable using this particular regimen.

The concept of using longer intervals and additional chemotherapy during the resting period was investigated and did result in significantly higher rates of pCR and cCR rates in patients with rectal cancer [45]. The additional cycles of chemotherapy delivered between RT completion and assessment of tumor response were labelled as consolidation chemotherapy and were originally designed to improve primary tumor response [45]. Compared to standard nCRT regimens achieving pCR and cCR rates of nearly 15–30%, nCRT with consolidation chemotherapy initially reported pCR and cCR rates in the range of 30–50% [46,47,48].

Shortly thereafter, the concept of using systemic chemotherapy preoperatively was expanded to address the possibility of treating micrometastasic disease upfront (in addition to improving primary tumor response) and potentially improving survival of patients [49]. This concept, named as total neoadjuvant therapy, incorporated systemic chemotherapy being delivered immediately before (induction) or after (consolidation) chemoradiation in an attempt to improve survival and primary tumor response [50]. Elegantly-designed randomized clinical trials clearly demonstrated that TNT regimens resulted in higher chances of achieving a pCR when compared to patients undergoing standard nCRT regimens [51].

However, when TNT regimens were compared between them, consolidation chemotherapy regimens (in the setting of long-course RT) were more likely to result in pCR or even cCR when compared to induction regimens (also in the setting of long-course RT) [52,53].

There is still a question as to whether short-course or long-course RT TNT regimens are better in achieving a cCR and leading to organ-preservation. No RCT using short-course RT used cCR as a primary endpoint for that purpose and there are ongoing trials comparing these 2 regimens head-to-head in the setting of consolidation chemotherapy (ACO/ARO/AIO-18.1 Trial; NCT04246684). Short-course RT would have the potential benefit of shorter treatment time (convenient for patients and allows more rational use of resources to deal with the considerable number of patients in need of treatment) [54]. However, little is known about the denominators of patients treated by short-course RT and consolidation chemotherapy that achieve a cCR and successfully undergo organ-preservation [55,56]. This contrasts with the recent estimates of nearly 50–60% of patients with locally advanced disease who avoid surgery in the setting of long-course RT and consolidation chemotherapy [53].

One important drawback of this treatment strategy using short-course RT has been recently reported in the setting of an RCT. Even though patients treated in the experimental arm had similar baseline staging features, underwent similar R0 resections and achieved a pCR 2× more frequently than the standard nCRT used in the control arm [55], local recurrence was significantly worse in the experimental arm [57]. One could argue that when using this approach to achieve a cCR, patients that eventually do not achieve a cCR may face higher chances of developing local recurrence even in the setting of an R0 resection.

All these studies have been performed for a population of patients with locally advanced rectal cancer—implying the oncological need for neoadjuvant therapy. However, studies have also reported on the outcomes of TNT regimens for cT2N0 disease. Apparently, patients with cT2N0 receiving TNT with long-course RT and consolidation chemotherapy have a significantly higher chance of achieving a cCR and avoiding TME [58].

More recently, nCRT regimens incorporating RT dose escalation techniques using contact radiation/brachytherapy (CxB) have been compared to standard nCRT in randomized trials using organ-preservation as their primary endpoint. Even though patients with cCR were grouped together with those achieving near-complete clinical response, organ-preservation rates were significantly higher in the experimental arm using CxB. While the ≥80% organ-preservation rate within the experimental arm in this study is quite remarkable, lack of widespread availability of the required CxB machine and expertise may considerably limit its implementation in clinical practice [59].

## 5. Assessment of Tumor Response

### 5.1. Assessment Tools

Assessment of tumor response to nCRT/TNT should be performed using the same assessment tools used at baseline assessment. Initial experiences with W&W were mainly derived from experience with clinical and endoscopic assessment tools, as radiological tools as we have available today were largely unavailable at that time [2,5].

### 5.2. Three-Pillar Assessment Criteria

Clinical assessment using DRE remains of critical relevance here. Findings consistent with a cCR include a smooth surface of the rectal wall at the area harboring the initial tumor and minimal induration of the rectal wall. There should be no ulceration, palpable mass or stenosis of the rectum [29]. It is the authors’ experience that, sometimes, subtle irregularities are best felt by DRE than seen on endoscopy or radiological imaging modalities. We strongly advise surgeons who assessed the primary cancer at baseline to perform reassessment for tumor response to provide best possible comparison. Clearly, it should be stressed that tumors beyond the reach of the finger during DRE should perhaps be considered suboptimal candidates for W&W.

**Figure 2:**
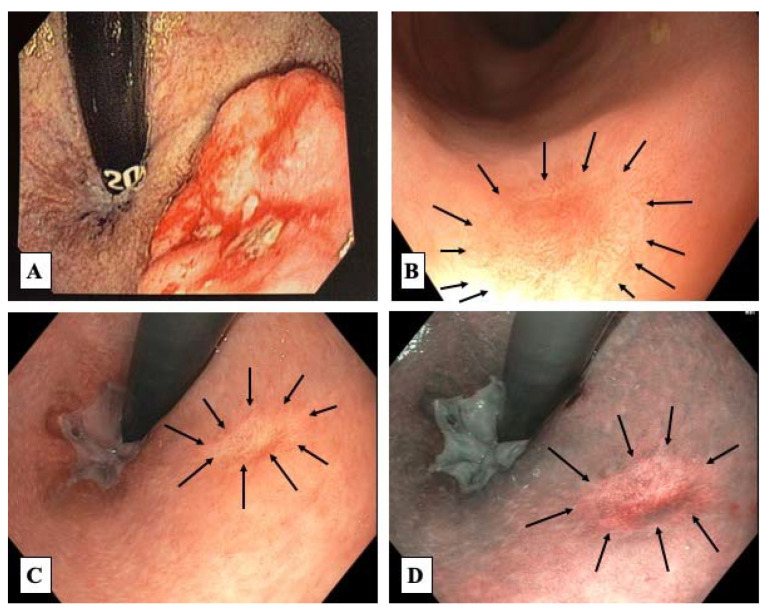
Endoscopic view of a baseline tumor (**A**) and post-treatment findings consistent with a cCR using direct view (**B**), retroflexive view (**C**) and narrow-band imaging (**D**). Throughout images (**B**–**D**), one can appreciate the presence of a white scar and significant telangiectasia (arrows). There are no ulcers or stenosis of the rectum.

Endoscopic assessment is the second pillar in reassessment of response to nCRT/TNT. Usually, flexible endoscopy is currently preferred in order to (1) provide documentation of the endoscopic appearance of the residual scar/tumor; (2) to allow advanced imaging techniques such as narrow band imaging and (3) to allow retroflexive view of the anal canal (commonly performed using the gastroscope rather than the colonoscope) and fully appreciate tumors close to anal canal and dentate line. Endoscopic findings consistent with a cCR include a white scar, no ulceration of the rectal wall or no irregularities of the mucosa. There should be no stenosis of the rectum allowing for a smooth progression of the colonoscope through the area harboring the primary tumor. While teleangiectasias are often detected within the area of the original tumor, irregular redness of the mucosa should be perceived as suspicious and preferably disappear in subsequent reassessments if this is the only positive finding during endoscopic assessment [29] (Figure 2).

**Figure 3:**
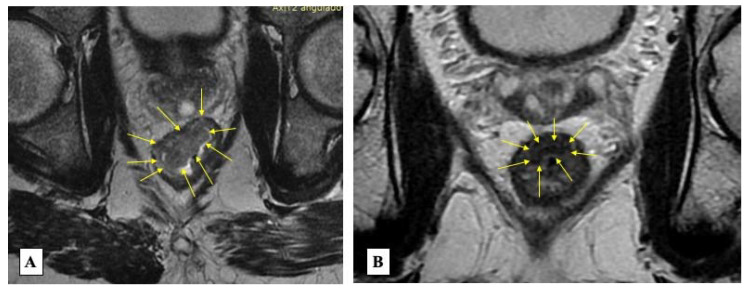
Radiological assessment using MR showing the baseline tumor (**A**—arrows) and an area of low-signal intensity areas consistent with a complete response in T2-weighted images (**B**—arrows).

The third pillar in reassessment is radiological imaging of the rectum, mesorectum and lateral pelvic compartment. Originally, imaging following neoadjuvant treatment was directed to assess almost exclusively the mesorectal and lateral pelvic compartment in the search for the presence of residual disease within lymph node, vascular structures (extramural vascular invasion—EMVI) and/or tumor deposits (replacement by tumor tissue within lymph nodes, blood and lymphatic vessels or nerves). However, as imaging techniques and interpretation have improved over the years, radiology can provide accurate information regarding the rectal wall itself. T2-weighted sequences often suffice for assessment of response without the need for intravenous contrast [60]. A proposed classification system has been commonly used to grade response (similarly to the pathological grading system; MRI Tumor regression grade - TRG) according to the presence of low-signal intensity areas. mrTRG1-2 are usually associated with complete or near-complete tumor response (suggesting a significant replacement by fibrotic tissue) in contrast with mrTRG3-5 [61]. Low-signal intensity areas may be regular or irregular and comparison with baseline imaging features may be helpful in assessment of response. While there is still controversy surrounding the usefulness of diffusion-weighted sequences [30,62,63,64], areas of restriction during this sequence may provide functional information and indicate the presence of residual cancer, adding to interpretation of T2-weighted sequences [65] (Figure 3).

PET–CT has been used for the purpose of tumor response assessment in several studies and may be useful for the identification of appropriate candidates for W&W in this setting [66,67,68]. However, as mentioned previously, reassessment by PET–CT preferably requires a baseline assessment using PET–CT. Considering the limitations in image resolution within this study (inferior to the high-resolution MRI images), considerable costs and the requirement of radiation associated with PET–CT (contrasting with MRI), the radiological study of choice has been high-resolution MRI in most cases.

Endoscopic biopsies are not included as one of the pillars for the diagnosis of a cCR. This means a negative endoscopic biopsy is not required to select patients for a W&W strategy if a cCR has otherwise been achieved [69]. This must be approached carefully, as surgeons often consider that an endoscopic biopsy is useless for the assessment of response. This is clearly not the case and often endoscopic biopsies may aid in the decision process in individual situations. In general, a negative endoscopic biopsy should not be considered as diagnostic for a complete response if there is INCOMPLETE clinical response. This is due to the low negative predictive value of this diagnostic tool in this setting (nearly only 20%). However, a positive endoscopic biopsy may be informative and useful in such patients with very significant yet incomplete responses (“near-complete” responses).

### 5.3. Timing

#### 5.3.1. First Assessment

Tumor response is a time-dependent phenomenon. Still, it appears that response is not linear over time and majority of response is usually observed early on either during or immediately after completion of radiation [31]. This means that patients who achieve a cCR will exhibit the majority of tumor regression early on during or after treatment [32,70]. Tumors with poor response after completion or 6 weeks from RT completion are unlikely to present major further response after such period [31]. In this setting, we recommend a first assessment of response between 6–8 weeks to attest significant response taking place early on as a sign of promising outcomes in achieving a cCR and successful W&W [34]. Because local regrowth is a risk among these patients, an early initial assessment of response may be quite useful. Many of these patients may actually present with excellent response at this very first assessment round and subsequently develop a local regrowth (which remains very small on the 2nd reassessment round). The fact that a first reassessment has been performed may allow for the correct identification of a local regrowth [34]. Had a first early assessment of response not been performed, this small early regrowth could have been mistaken for a near-complete ongoing response leading to further delay in diagnosis and subsequent definitive treatment of the regrowth.

#### 5.3.2. Reassessment Rounds

Even though the majority of response is observed within early time intervals from RT completion, it may actually take longer intervals for tumors to achieve all strict criteria for a cCR. One retrospective study indicated that the majority of cCR were only achieved after 16 weeks from RT completion in patients receiving CRT and consolidation chemotherapy [33]. Therefore, one has to consider that many of these patients will present with significant yet incomplete clinical responses in one or more of the reassessment rounds. It has been suggested that whenever patients achieved a near-complete clinical response, subsequent reassessment could lead to achievement of cCR in a rather high proportion of patients [33]. However, the “near-complete” response is a poorly-defined clinical entity with very little consensus in its definition [71]. Instead of labelling patients to harbor “near-complete” clinical response, the readership may perhaps consider the presence of the following features in deciding for subsequent reassessment of response rounds prior to definitive surgical resection of the primary:

First, tumors should have exhibited significant response in their very first assessment round. Comparison with baseline assessment information may be helpful in identifying patients who truly exhibit very significant responses (nearly 75–80% of the tumor volume is gone by endoscopic assessment and only a minor irregularity is detected) [32]. One should be careful, as small tumors that remain small may be less responsive when compared to large baseline tumors that nearly disappear. DRE should be questionable and only minor irregularities should be accepted. Radiology should be identifying patients with mrTRG1-2 only [61].

Second, there should be ongoing response in between rounds, meaning that there is no stable incomplete clinical response. Instead, clear subsequent regression needs to be clearly documented with any of the assessment studies [33].

Third, endoluminal response seems to be the driver of response. This means that endoscopy and DRE showing complete disappearance of the tumor should be considered more significant than radiological disappearance of the disease. In other words, if there is no evidence of cancer during DRE and endoscopy, even if there is suspicious residual cancer on MRI, we believe a subsequent reassessment may be harmless and offer the opportunity for achievement of all criteria of cCR (including radiological). However, patients with mrTRG1 and the presence of ulceration with elevated borders and highly suspicious areas palpable during DRE should probably undergo resection of the primary disease.

Finally, most cCR are usually achieved within 6 months from RT completion [33]. If incomplete response is still obvious after 24–26 weeks from RT completion, surgical resection of the primary is perhaps preferred [72].

## 6. Local Regrowth

### 6.1. Definition and Salvage

**Figure 4:**
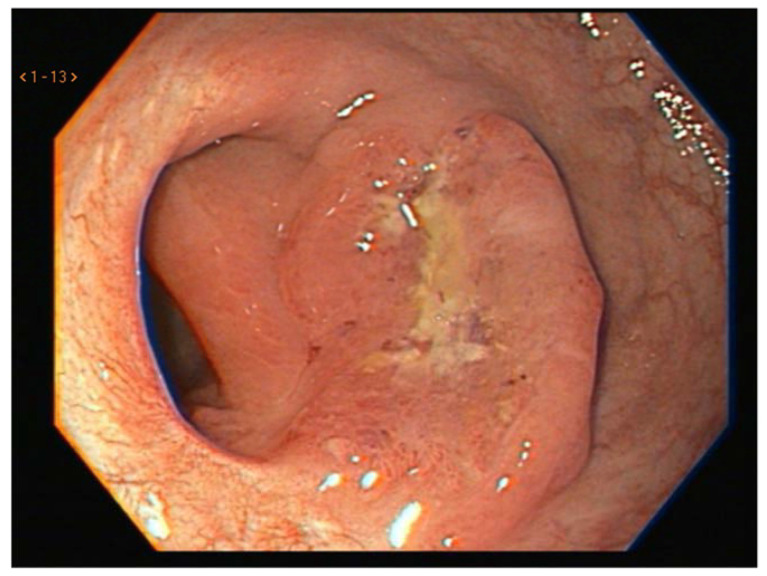
Endoscopic view of a local regrowth following the achievement of a cCR.

Local regrowth is, by definition, the reappearance of the primary tumors within the rectal wall, the mesorectum or within the lateral pelvic compartment after the achievement of a cCR [73]. It seems that nearly 25–30% of patients who achieve a cCR and are managed non-operatively will eventually develop a local regrowth [74]. This risk is highest within the 3 years immediately after the achievement of a cCR [6,18,19,74] (Figure 4).

The term regrowth instead of recurrence was originally proposed to distinguish this clinical entity from local recurrences after TME surgery. The idea was to attempt to avoid the stigma associated with local recurrences following TME frequently associated with poor outcomes, often unresectable and frequently associated with debilitating condition [73]. Instead, local regrowth is, in the majority of the cases amenable to salvage resection, possible through an R0 resection in nearly 90% of the cases [75,76,77,78]. In fact, surgical salvage of local regrowth provides excellent local disease control with subsequent re-recurrence in ≤5% of the cases. A significant proportion of patients requiring salvage TME at the time of local regrowth ultimately require an abdominal perineal resection (APR) [79]. In fact, the rates of APR among regrowth seem to be higher than in patients proceeding straight to TME after treatment completion and incomplete clinical response [78]. These differences may be due to distinct features between patients being offered W&W versus those undergoing TME related to tumor location. Since ideal candidates for W&W are those with tumors located at the reach of the finger during DRE and located at the level of or below the insertion of the levator ani muscles, it is not surprising that local regrowth at this level would frequently require APR [80].

Still, a proportion of these patients may also be salvaged by a second opportunity for organ-preservation: transanal local excision of the regrowth. This has been recently reported in two independent series. Curiously, disease-free survival among local regrowth salvaged by local excision was better than patients undergoing TME for salvage. However, locally-excised regrowth was more likely to have early-stage disease at baseline. Therefore, such differences in survival may possibly be reflecting intrinsic differences in baseline stages rather than the actual type of salvage employed at the time of regrowth [81,82].

### 6.2. Risk Factors

Risk factors for development of local regrowth after the achievement of a cCR appear to be related exclusively to baseline T stage [83]. Apparently, there seems to be a 10% increase in the risk of local regrowth for every increase in T stage category: 20% for cT2, 30% for cT3 and 40% for cT4 [18]. Curiously, baseline N stage has not been associated with the risk of a local regrowth in these patients [84]. Interestingly, when patients sustain a cCR longer than 3 years, the risk of local recurrence becomes minimal. In addition, risk factors such as baseline T stage become irrelevant. This suggests that the main driver in the risk of local regrowth is primary response to treatment, overriding risk factors associated with baseline features or even treatment-related [85].

## 7. Distant Metastases

The risk of distant metastases among patients who achieve a cCR after treatment is considerably low [74]. In fact, an early report suggested that this risk was considerably higher among patients who developed local regrowth compared to those who did not [86]. In fact, subsequent studies examining larger datasets of patients with cCR looked for risk factors for development of distant metastases after entering a W&W program. Curiously, the only identifiable risk factor for development of distant metastases in this series was previous development of local regrowth [75,87]. However, one has to consider that these patients are biologically intrinsically different: cCR who never develop local regrowth are true complete responders; whereas local regrowth are necessarily incomplete responders that were mistaken for a cCR [88]. Therefore, it is not surprising that they have distinct risks for development of distant metastases.

However, the risk of distant metastases after cCR becomes very low after completion of 5 years of follow-up from the baseline cancer. Instead, local regrowth sustains a considerable risk for distant metastases until they complete 5 years, not from the baseline cancer but from the local regrowth [85,87]. This suggests that leaving the incomplete response in situ for variable periods of time may increase the risk of distant metastases already provided by the baseline cancer.

In order to address this question, patients with local regrowth managed by salvage resection at the time of local regrowth have been compared to incomplete responses managed by straight surgery after completion of treatment. There were no differences in survival both in retrospective and prospective series [53,79]. Still, such comparison may also not be fair since local regrowth had excellent initial response to treatment (it was even mistaken for a cCR). The control group (TME straight after treatment) did not necessarily have excellent response. Therefore, one could argue that local regrowth that was salvaged should have had better survival outcomes than all TMEs.

In this setting, an even more recent study compared the outcomes of salvage surgery for local regrowth to the outcomes of TME straight after treatment in the setting of excellent tumor response (to balance for response to treatment across groups). In this study, two independent risk factors were identified: local regrowth and ypT3-4 in the resected specimen. Combination of both features—local regrowth and ypT3-4—in the resected specimen led to significantly worse distant-metastases-free survival. This suggests that patients who have excellent (but incomplete) response managed by TME straight after treatment respond better than those undergoing salvage TME at the time of local regrowth. (Dis Colon & Rectum—accepted for publication—in press).

While this is restricted to a very small proportion of patients who achieve a cCR, patients need to be fully aware of the risks associated with W&W in this setting.

## 8. Surveillance Strategy

Considering the risk of local regrowth is most frequent within 3 years of follow-up after the achievement of a cCR, and the risk of subsequent distant metastases among this subgroup of patients, surveillance has been adapted to attempt to minimize these risks [85].

First, surveillance is more intensive during the first 3 years of follow-up. Usually, these patients are assessed for their primary tumors every 8 weeks. Second, surveillance is recommended for life even though the risk of regrowth becomes very low after 3 years being disease-free. Third, we want to avoid the risk of detecting a local regrowth harboring ypT3-4 disease within the resected specimen. Taking all this information into account, patients should preferably undergo a DRE, proctoscopy, CEA levels and MRI every 8–12 weeks during the first 3 years. After these 3 years, follow-up may perhaps be loosened to every 6 months and to include primary tumor response assessment [34]. Metastatic disease surveillance should follow the usual guidelines [89] and should only be intensified in the case of (1) a local regrowth and/or (2) clinical suspicion. After the 5th year, patients can follow-up yearly.

Ultimately, this surveillance program results in frequent visits required for clinical, endoscopic and radiological assessment particularly during the first 3 years of follow-up. Patients should be clearly and fully informed upfront of this recommended follow-up and understand the potential clinical and oncological consequences of local regrowth. Failure to comply with appropriate follow-up in a W&W program may constitute a relative contra-indication for any organ-preservation strategy. Still, surveillance strategies may differ across different institutions and have changed over time. Therefore, in line with any surveillance strategy for rectal cancer regardless of definitive surgical or non-surgical management, patients are not specifically asked to consent to a fixed surveillance program.

## 9. MSI Rectal Cancer and Watch and Wait

Patients with high-level microsatellite instability (MSI-high) adenocarcinomas were found to have worse rates of complete response to standard nCRT [36]. The development of PD1 checkpoint inhibitors led to the observation of significant tumor response in multiple tumors with MSI status. The idea is that activation of a specific immune response is capable of eliciting significant anti-tumoral effect of the host immune system, eventually leading to a significant proportion of complete response in multiple primary solid tumors. One recent single-arm clinical trial including primary MSI-high rectal adenocarcinoma offered PD-1 checkpoint inhibitors leading to a surprisingly 100% cCR rate among 14 patients, without the use of radiation or standard chemotherapy [7]. While these exciting and enthusiastic results await further longer follow-up and increase in the number of patients included, it creates a clear distinction based on molecular features of rectal cancers dependent on microsatellite instability and the possibility of treatment with immunotherapy potentially leading to avoidance of TME in the vast majority (if not all!) patients.

## 10. Conclusions

In conclusion, Watch & Wait is now considered as an attractive alternative to TME for patients with distal rectal cancer. Patients who achieve a cCR using strict clinical, endoscopic and radiological criteria may benefit from avoiding radical surgery when surgical alternatives are APR or intersphincteric resections. Functional outcomes after W&W appear to be superior to TME in this setting. Careful surveillance should be recommended for early detection of tumor regrowth, which appears to be observed in nearly 30% of patients entered in a W&W program within 3 years of completion of treatment. Salvage surgery seems to be safe for local regrowth and provides good long-term local disease control. The risk of distant metastases is overall low among patients undergoing W&W. However, the subset of patients who develop local regrowth may be at higher risk for developing subsequent metastatic disease.

## Data Availability

Not applicable.
